# The Length of the Transition Zone in Patients with Rectosigmoid Hirschsprung Disease

**DOI:** 10.3390/children9020152

**Published:** 2022-01-25

**Authors:** Christian Tomuschat, Stefan Mietzsch, Sebastian Dwertmann-Rico, Till Clauditz, Hansjoerg Schaefer, Konrad Reinshagen

**Affiliations:** 1 Department of Pediatric Surgery, University Medical Center Hamburg-Eppendorf, 5220246 Hamburg, Germany; s.mietzsch@uke.de (S.M.); k.reinshagen@uke.de (K.R.); 2 Department of Pathology, University Medical Center Hamburg-Eppendorf, 5220246 Hamburg, Germany; s.dwertmann-rico@uke.de (S.D.-R.); t.clauditz@uke.de (T.C.); hansjoerg-schaefer@t-online.de (H.S.)

**Keywords:** Hirschsprung disease, transition zone, aganglionic zone

## Abstract

**Background:** The transition zone (TZ) is defined by specific histological findings in patients with Hirschsprung Disease (HSCR). HSCR treatment includes surgical removal of the aganglionic zone (AZ). During the pull-through procedure, it is critical to resect the TZ. Given the TZ’s wide histological heterogeneity, we wanted to know how extensive the histological transition zone is. **Methods:** A retrospective study of patients who had pull-through surgery for rectosigmoid HSCR between January 2010 and December 2020 was carried out. Demographics, length of TZ and AZ, age and symptoms upon presentation, and complications after surgery were also obtained. **Results:** The inclusion criteria were met by 50 patients. The mean age of all patients was 10 months (0.1–107.5 months), with a mean age at pull-through of 16.3 months (3–112 months). Thirty-one out of fifty patients (62%) received primary laparoscopic endorectal pull-through surgery (LEPT). The average TZ length of all patients was 2.6 cm (0–10 cm), and the AZ length was 9.6 cm (1–30 cm). The length of the AZ and TZ were shown to have no correlation (r² = 0.237). **Conclusions:** The current study found that the mean length of the TZ in individuals with rectosigmoid HSCR is less than 5 cm in most cases and has no correlation with the length of the AZ.

## 1. Introduction

Hirschsprung disease (HSCR) is distinguished by a lack of ganglion cells in the distal bowel, which may extend proximally to varying lengths [1]. A transition zone (TZ) is almost always seen proximal to the colon’s aganglionic segment (AZ) and distal to the histologically identifiable ganglionic section. This TZ, like the aganglionic portion, varies in length. Most characteristic histological findings, however, are frequently restricted to a 3–5 cm long zone proximal to the aganglionic segment [2,3]. The TZ is characterised by a reduction in the number of ganglion cells in the submucosa and muscularis propria, as well as more irregular and sparse ganglion cells with partially broadened nerve fibre bundles [3]. Hypoganglionosis is seen in slightly discontinuous sections of the TZ’s circular colonic plane. Indeed, the unequal circumferential distribution of ganglion cells in both the myenteric plexus and the transition zone submucosa results in a “leading edge” of ganglion cells extending into the aganglionic distal bowel, which occasionally confuses pathohistological diagnosis [4]. Giant ganglia cells have been found on rare occasions in our experience.

According to Kapur et al., the most common definitions for the TZ include circumferential aganglionosis, myenteric hypoganglionosis, and submucosal nerve hypertrophy [5].

After being diagnosed with HSCR, patients will need a pull-through operation, which is commonly performed during the neonatal period or within the first few months of life [5]. At our facility, we try to execute all surgeries at three months of age and at a weight of five kilograms. The procedure can be performed using either a transanal-only method or a laparoscopy-assisted approach. The latter has the advantage of standardised full-thickness histological mapping and the ability to mobilise the colon if necessary [6]. There is also evidence that a laparoscopic-assisted approach minimises the risk of anal overstretching compared to a transanal-only approach, with perhaps fewer postoperative problems due to obstruction [7]. However, persistent postoperative obstructive symptoms have been linked to inadequate TZ pull-through (TZPT) after surgery. The prevalence of a TZPT has been observed to range between 6 and 19 percent [8].

The TZ is typically 5 cm long; however, publications have demonstrated that the TZ can reach significantly longer lengths [5,9]. As a result, guidelines urge that the colorectal anastomosis should be at least 5 cm proximal to the most distal biopsy showing ganglion cells [3,4]. Surgery is a life-saving procedure for patients with HSCR. Since the likelihood of postoperative distal obstructive symptoms exists, failure to resect the TZ is one explanation for persistent postoperative symptoms [1,5]. We anticipate that the findings of this study may aid in the treatment of HSCR by assisting with intraoperative and postoperative surgical pathology.

## 2. Materials and Methods

**Figure 1:**
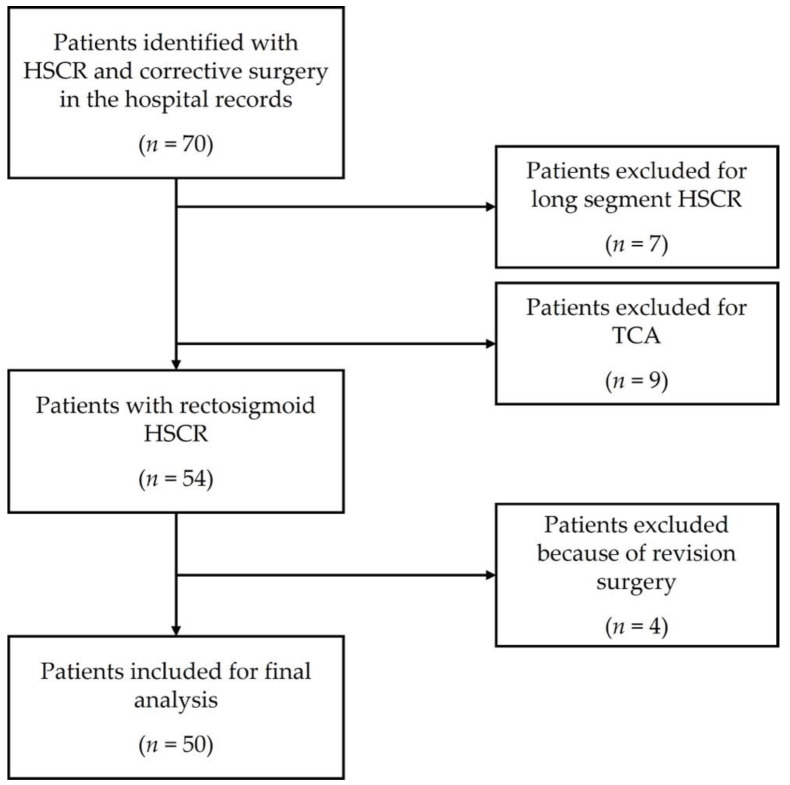
Patients included in the study.

**Figure 2:**
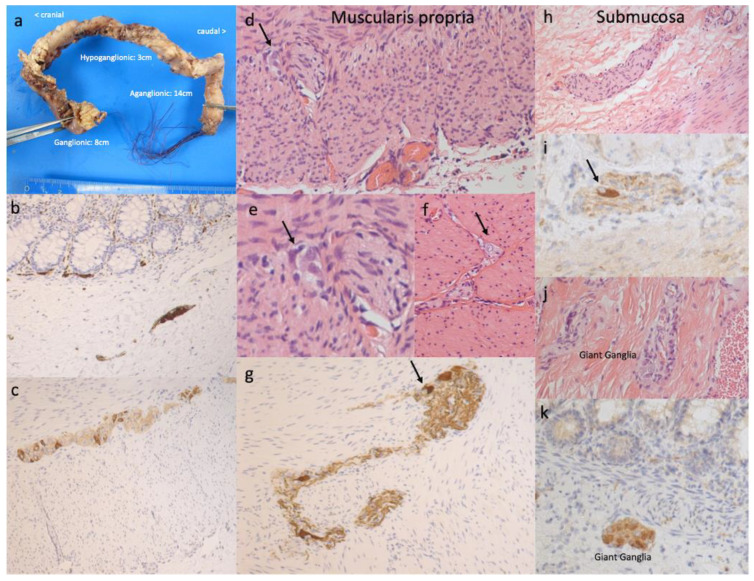
Pathomorphological aspects of the transition zone: (**a**) full specimen (**b**) and (**c**) ganglia in the submucosa and muscularis propria in normal colon stained with Calretinin or RET1. (**d**) Reduced number of ganglia in the muscularis propria (**e**) and (**f**) focal, irregular residual ganglia cells. (**g**) Calretinin staining of reduced ganglia cells. (**h**) Multifocal accentuated hypoplasia of ganglia cells and thickening of nerve fibres. (**i**) Reduced/irregular residual ganglia cells (**j**,**k**) Giant ganglia cells.

The study was approved by the Hamburg Ethics Committee (WF-200/20, 28.06.2021) and was in line with the Declaration of Helsinki and its later amendments. After receiving ethics approval, we conducted a retrospective chart review of all patients diagnosed with HSCR who underwent surgery between January 2010 and December 2020 at our paediatric surgery department. Exclusion criteria were (1) long-segment HSCR, (2) total colonic aganglionosis (TCA), and (3) revision surgery (Figure 1). Only patients with rectosigmoid disease were included since only a few patients with reliably mapped TCA were available. The length of the transition zone was determined by histological analysis of representative full-thickness specimens according to the established criteria (circumferential aganglionosis, myenteric hypoganglionosis, and submucosal nerve hypertrophy, Figure 2). Two researchers collected data, including general patient demographics and two-staged surgery, complications, and revision surgery. At our clinic, the surgical method of choice for HSCR was a laparoscopic-assisted pull-through at three months of age. The biopsies were then sent to the pathology department, which analysed the biopsies immediately. While awaiting the review, the transanal pull-through was commenced. Before the colorectal anastomosis, results were obtained by the pathology department, enabling conduction of the anastomosis at the appropriate location.

After surgery, the pathology department analysed all resected samples, and the aganglionic and transition zone lengths were formally reported. The pathohistological analysis in biopsies included frozen section diagnostic and, in some cases, in the resection, material immunohistochemical demonstration of ganglion cells by reactions on RET1 and Calretinin. The length of the TZ was characterised by established criteria [9,10,11]. Pearson correlation was used to determine the relationship between TZ and AZ. Kruskal–Wallis tests for age at diagnosis, age at surgery, and length of the transition or aganglionic zone were used to calculate the differences. For simple descriptive statistics, a paired T-test was used. All data were analysed with Prism 9.0.0. (GraphPad Software, LLC, San Diego, CA, USA).

## 3. Results

Of the 50 patients included, the overall male-to-female ratio was 2.5:1. Of all patients, 19 patients needed a stoma before the definitive pull-through procedure (38%). The overall mean age of all patients was ten months (0.1–107.5 months), with an overall mean age at pull-through of all patients at 16.3 months (3–112 months). Thirty-one out of fifty (62%) had undergone primary laparoscopic endorectal pull-through (LEPT) (Table 1).

**Figure 3:**
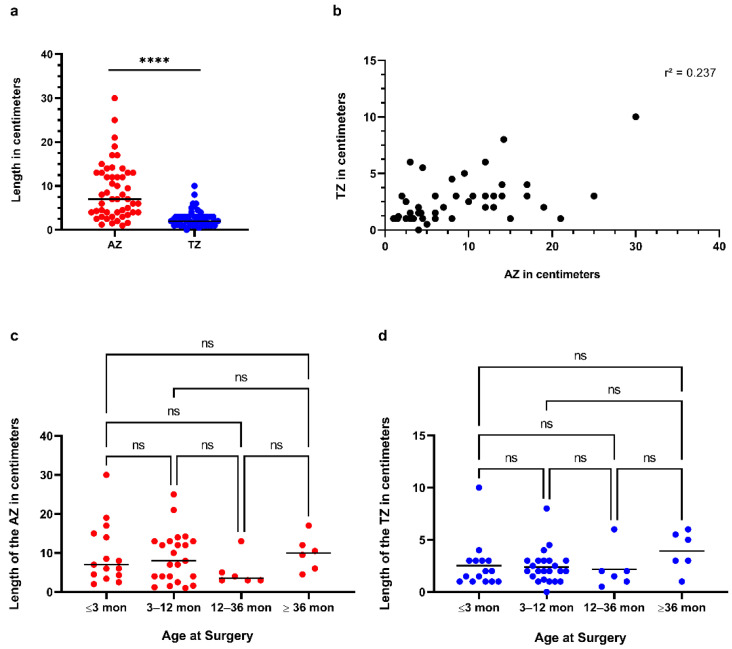
(**a**) Comparison between the transition and aganglionic zone lengths in centimetres of all patients showed a significant difference in length (*p* < 0.0001, “****”) (**b**) The relationship between the aganglionic zone and transition zone indicates no or negligible correlation between the length of the age at surgery and length of the transition or aganglionic zone in centimetres, represented by an r^2^ of 0.237. (**c**,**d**) A Kruskal–Wallis test between patients showed no significant difference in length and age at surgery.

**Figure 4:**
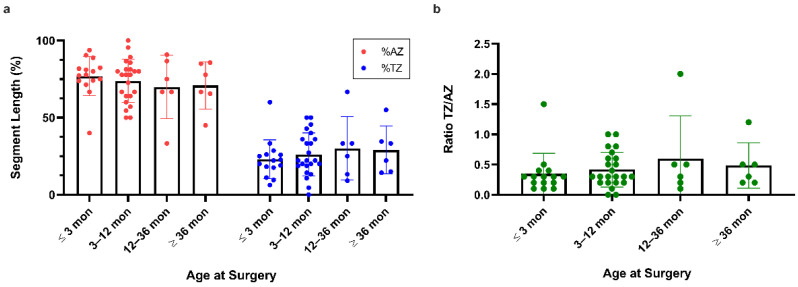
(**a**) Segment length of AZ and TZ in relation to the total length of the resected section sorted by age at surgery. The AZ section represents the major part of the resected bowel in most patients with a mean AZ = 73.97% and a mean TZ = 26.03%. (**b**) TZ/AZ ratio of all patient specimens sorted by age at surgery, showing that the TZ length is less than half the length of AZ in most patients (AZ/TZ ≤ 0.5 in *n* = 39 patients) and with a tendency towards a longer TZ in older patients.

**Figure 5:**
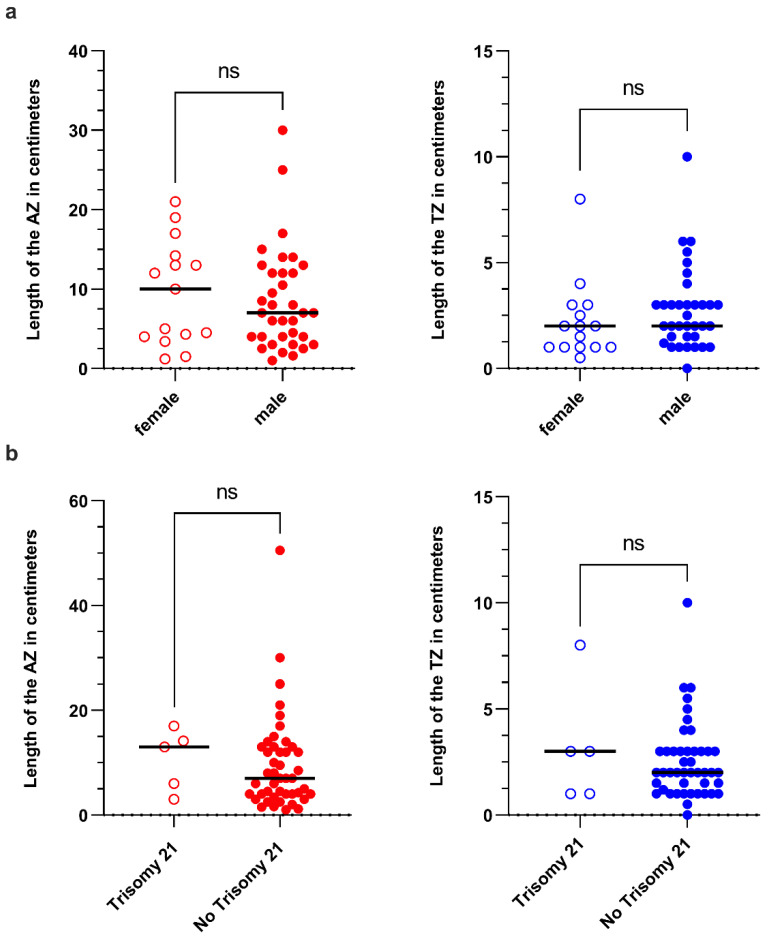
Analysis of the segment length of the AZ (**left**) and TZ (**right**) in centimetres in all patients regarding (**a**) gender and (**b**) presence or absence of Trisomy 21 revealed no significant difference between the groups.

The mean length of the TZ of all patients was 2.6 cm (0.0–10.0 cm) and of the AZ 8.9 cm (1.0–30.0 cm), respectively (Figure 2a). Only five patients had a TZ > 5 cm: two patients diagnosed at <1 months and three patients diagnosed at >12 months. Comparison between the transition and aganglionic zone lengths in centimetres of all patients showed a significant difference in length (*p* < 0.0001) (Figure 3a). The relationship between the aganglionic zone and transition zone indicates no or negligible correlation between the length of the aganglionic and transition zone, represented by an r^2^ of 0.237 (Figure 3b). The Kruskal–Wallis test between age at surgery and length of the transition or aganglionic zone in centimetres showed no significant difference in the length and age at surgery (Figure 3c,d). When we compared the proportions of AZ and TZ in different subgroups to the age at surgery, we discovered a positive trend toward patients who were operated on at a later age. In the ≤3 months age group, the AZ had a mean proportion of 76.93% (SD 12.57%, *n* = 15), while the TZ had a mean proportion of 23.03% (SD 12.56%). The AZ proportion in the age range 3–12 months was 73.87% (SD 13.90%, *n* = 23), while the TZ proportion was 26.143% (SD 13.89%, *n* = 6). The AZ proportion was 69.88% (SD 20.54%, *n* = 6) and the TZ proportion was 30.12% in the age range 12–36 months (SD 20.54%). The AZ proportion was 70.95 (SD 15.38%, *n* = 6) and the TZ proportion was 29.05% (SD 15.38%) in the eldest group (>36 months). The observed differences between subgroups were not statistically significant (Figure 4a,b). There were no gender differences in the length of the TZ or AZ nor significant differences in those with Trisomy 21 compared to the other HSCR patients (Figure 5).

Subgroup analysis revealed that nineteen patients presented during the neonatal period, of which four (21%) needed a stoma before final diagnosis due to distal bowel obstruction and not responding correctly to washouts. One patient presented with a bowel perforation at the colon ascendens. The mean age at diagnosis in that group was 0.4 months, and the mean age at pull-through was 4 months. The mean TZ in that group was 3.1 cm (1.0–10.0 cm), and the AZ was 11.0 cm (1.0–30.0 cm). Sixteen patients were diagnosed between 1–6 months of age with a mean age of 2.4 months. The mean age at a pull-through was 4.4 months. The mean length of the TZ was 2.2 cm (0.0–6.0 cm), and the AZ was 6.5 cm (1.5–15.0 cm). Four out of sixteen (25%) patients needed a stoma before the definitive pull-through procedure. Stoma placement surgery was most often indicated due to ileus and gross abdominal distension, which did not respond to washouts. Five patients were diagnosed between 6–12 months with a mean age of 9.0 months. The mean age at pull-through was 11.4 months. The mean length of the TZ was 1.3 cm (0.5–2 cm) and of the AZ was 9.5 cm (1.6–21 cm). Three out of five (60%) of the patients needed a stoma before the definitive pull-through procedure due to distal bowel obstruction and refractory obstipation. Ten patients were diagnosed beyond 12 months of age with a mean age of 41.2 months. The mean age at pull-through was 45.6 months. The mean length of the TZ was 3.0 cm (1.0–6.0 cm) and of the AZ 8.3 cm (3.0–17.0 cm). Eight out of ten patients needed a stoma due to abdominal distension, distal bowel obstruction, and failure to thrive.

Ten percent of the patients were diagnosed with Trisomy 21, which was also the most common syndromic association. Out of 50 patients, 2 presented in a familial context. One of these patients was born prematurely (31 weeks gestational age) and had a brother already diagnosed with HSCR. Four percent of the patients presented with signs of HAEC, one pre- and one postoperatively. Twelve patients needed Botox injections postoperatively. Following the laparoscopic-assisted pull-through, we had only one TZPT.

## 4. Discussion

In this study, the average TZ length was 2.6 cm. The longest TZ measured was 10.0 cm, with just 5/50 patients having a TZ longer than 5 cm. These findings are in line with previous research [2,4,10]. Furthermore, we were unable to find any substantial variation within this margin or to link potential differences in diagnosis or surgery age to TZ length (Figure 3c,d). We computed the TZ proportion due to the projected longitudinal extension of the colon, which may also complicate the interpretation of intestinal lengths at different ages. The length of TZ relative to AZ appeared to show an increasing trend with age. However, this was not statistically significant and actually decreased in the oldest cohort (Figure 4a,b). Furthermore, there was no difference in TZ length and gender (Figure 5a). In addition, we did not find a difference in TZ length for individuals with Trisomy 21, which could better explain this subgroup’s poorer result (Figure 5b). However, because we only had five (10%) individuals with Trisomy 21 in our group, the data should be interpreted with caution.

The majority of the three major TZ characteristics (partial circumferential aganglionosis, myenteric hypoganglionosis, and submucosal nerve hypertrophy) were limited to bowels less than 5 cm from the AZ. Although there was a positive trend in the length of TZ, there was no correlation between the AZ and TZ in the current cohort (r^2^ = of 0.237, Figure 3b). Longer TZ may become increasingly common with long-segment HSCR or TCA as a result of this trend. However, larger cohorts are required to validate these findings. According to the current literature and our findings, performing the coloanal anastomosis 5–10 cm proximal to the most distant ganglionic biopsy is safe [4,5]. Ninety percent of HSCR patients will be diagnosed during their newborn period [6]. It was discovered in our cohort that only 38% were diagnosed within the first four weeks of life, 32% within the first eight weeks of life, 10% within one year, and 20% thereafter, with a mean of 41 months. This is in contradiction to what has been published. There is a mechanism that could explain the contradictory results. First and foremost, we serve as a referral centre for other hospitals. Some of the later-diagnosed patients may have had neonatal symptoms, and the late diagnosis was caused by iatrogenic factors. This was especially true for patients who were diagnosed more than a year after being referred. This was also seen in the increased stoma rate among individuals diagnosed later in life (60% and 80%, respectively). We are in the range of previously reported data, with a total of 38% of the stoma rate [12]. However, the rate increased as patients were diagnosed later in life (Table 1). Due to the delayed manifestation of HSCR, individuals may have significant colonic dilatation. At our clinic, we open an ileostomy to help decompress the proximally dilated colon. We believe that a small subset of patients with late-onset HSCR may benefit from primary LEPT surgery; however, in most cases, a diversion via an ileostomy is the safest option while also avoiding potential complications during and after anastomosing a dilated colon [13]. Once HSCR is verified, we typically perform an LEPT at the age of three months and at a weight of five kilograms, if the patient is stable. The use of laparoscopic assistance enables colon mapping prior to the transanal operation. In addition, if necessary, the left colon can be mobilised. As a result, the study only found one TZPT (2%) at our clinic in the last ten years. One reason for the low incidence of TZPT is our policy of performing the coloanal anastomosis 5 cm proximal to the most distal ganglionic biopsy and using laparoscopic mapping of the colon to identify the exact length of the TZ in all HSCR patients. The prevalence of TZPT has been reported to range between 6% and 19% in the literature [8]. The upper margin of the reports appears to be rather high to us. The incidence of TZPT is low at experienced centres and should be about 5%.

Furthermore, only 4% of our patients had signs of enterocolitis (Hirschsprung-associated enterocolitis—HAEC); one patient had HAEC before and one after the PT operation. Although the molecular causes of enterocolitis are not entirely understood, in our experience, faecal stasis due to obstruction is usually the major cause of developing HAEC [14]. The primary treatment options for distal bowel obstruction are (1) thorough rectal washouts as needed and (2) stoma surgery and placement proximal to the obstruction. Most paediatric surgeons recommend rectal washouts followed by primary transanal PT surgery [15]. When rectal washouts are inadequate in relieving the obstruction or the patient is not ready for surgery, two-staged surgery is a safe and reasonable option.

## 5. Limitation

The current study may be limited in accuracy due to missing data in the charts due to the study’s retrospective methodology. There may also be a lack of uniformity because the pathological specimens were examined by two separate paediatric pathologists. However, because both pathologists operate at the same institution and use the same analytical criteria, the inaccuracy is decreased. Since postoperative clinical follow-up was anticipated to be insufficient and brief in many patients, we opted not to include those data in the current investigation.

## 6. Conclusions

The study cohort of 50 rectosigmoid HCSR patients indicated that the TZ does not correlate with the length of the measured aganglionic part. The TZ measured 2–5 cm in 95% of the specimen, supporting the former recommendation to perform a pull-through 5–10 cm cranially from the most cranial ganglion cell exhibiting biopsy.

## Figures and Tables

**Table 1:** Demographics of the patients included in the study.

Characteristic			*n* (%)
Sex			
Male			35 (70%)
Female			15 (30%)
Age at Diagnosis in Months			
<1			19 (38%)
1–6			16 (32%)
6–12			5 (10%)
>12			10 (20%)
	*Mean Age at Diagnosis*	*Mean Age at Pull-Through*	
<1	0.4	4.0	
1–6	2.4	4.3	
6–12	9.0	11.4	
>12	41.2	45.6	
	*Mean Length of TZ in cm*	*Mean Length of AZ in cm*	
<1	3.1	11.0	
1–6	2.2	6.5	
6–12	1.3	9.5	
>12	3.0	8.3	
Pull-Through Procedure			
Primary			31 (62%)
Two-Stage (Stoma)			19 (38%)
Mean Age Distribution for Two-Stage (Stoma) in months			
<1			4/19 (21%)
1–6			4/16 (25%)
6–12			3/5 (60%)
>12			8/10 (80%)
Nonsyndromic			41
Syndromic			9
Trisomy 21			5
Others			4
Familial			2
